# Lower limb injury prevention programs in youth soccer: a survey of coach knowledge, usage, and barriers

**DOI:** 10.1186/s40634-018-0160-6

**Published:** 2018-10-11

**Authors:** Robin Mawson, Michael J Creech, Devin C Peterson, Forough Farrokhyar, Olufemi R Ayeni

**Affiliations:** 1 Faculty of Medicine, University of Toronto, Toronto, ON Canada; 2 North Bay Regional Health Centre, North Bay, ON Canada; 3 Division of Orthopaedic Surgery, Department of Surgery, McMaster University, Hamilton, ON Canada; 4 Department of Surgery, McMaster University, Hamilton, ON Canada; 5 Department of Epidemiology and Biostatistics, McMaster University, Hamilton, ON Canada; 6 Department of Health Research Methods, Evidence and Impact, Hamilton, ON Canada

**Keywords:** Injury prevention, Coaches, Soccer, Youth sports, Child, Adolescent, Warm-up exercise, Anterior cruciate ligament, Lower extremity

## Abstract

**Background:**

Participation in youth soccer carries a significant risk of injury, most commonly non-contact injuries of the lower extremity. A growing body of research supports the use of neuromuscular interventions by teams to prevent such injuries, yet the uptake of these recommendations by soccer teams remains largely unexplored. The purposes of the study were to determine (1) the level of awareness by youth coaches of injury prevention programs and their efficacy; (2) the number of youth coaches that use these interventions; and (3) barriers and potential facilitators to implementing a sustainable injury prevention program.

**Methods:**

Four hundred eighteen coaches of male and female youth soccer teams were emailed an online blinded survey. This survey consisted of 26 questions covering coaches’ demographics, level of training, experience with injuries among players, and use of injury prevention programs. Question development was guided by the RE-AIM Sports Setting Matrix in combination with findings from the literature review and expert experience from orthopaedic surgeons specializing in sport medicine.

**Results:**

Of the 418 coaches contacted, 101 responded. Only 29.8% of respondents used an injury prevention program in the prior soccer season. Coaches that had completed one or more coaching courses were more likely to use an intervention. Of those that did not already use an intervention, coaches agreed or strongly agreed that they would consider using one if it could be used in place of the warm up and take no more than 20 min (74.0%), if they could access information about the exercises (84.0%), and if the exercises could be properly demonstrated (84.0%). Additionally, 84% of coaches that did not already use an intervention agreed or strongly agreed that knowing that interventions may reduce a player’s risk of injury by 45% would affect whether they would use one.

**Conclusion:**

This study suggests that the current use and awareness of injury prevention programs is limited by a lack of communication and education between sporting associations and coaches, as well as perceived time constraints. The results also suggest that improving coaching education of injury prevention could increase the frequency of intervention use.

**Electronic supplementary material:**

The online version of this article (10.1186/s40634-018-0160-6) contains supplementary material, which is available to authorized users.

## Background

Soccer is the world’s most popular sport, with nearly 300 million players world-wide (FIFA big count 2006, 2007). Unfortunately, soccer participation is associated with a significant risk of lower limb injury and has a higher incidence of injury than many contact or collision sports (Koutures & Gregory, 2010; Radelet et al., 2002; Wong & Hong, 2005). The most common injuries in soccer players are non-contact injuries of the lower extremity (Wong & Hong, 2005). Male players are more likely to sustain injuries to their ankles while female athletes are more prone to injuries to their knees (Radelet et al., 2002; Wong & Hong, 2005; Le Gall et al., 2008). The US Consumer Product Safety Commission National Electronic Injury Surveillance System has data to suggest that many of these injuries (over 80%) occur in players under the age of 24 (NEISS Data Highlights - 2015, 2015). Anterior cruciate ligament (ACL) tears are a common example of a lower extremity injury from soccer. ACL injuries occur most commonly in non-contact sports that involve pivoting, quick accelerations and decelerations, and jumping (Alentorn-Geli et al., 2009; Boden et al., 2000), making soccer players prime candidates for these injuries.

Many sports associations have recognized the risk of injury for young soccer players, and have developed injury prevention programs, or interventions, in order to help reduce them (Bizzini et al., 2013; Hübscher & Refshauge, 2013). The Canadian Academy of Sport and Exercise Medicine has developed a position statement and made recommendations to implement injury prevention programs in youth soccer players in order to decrease the incidence of ACL injuries (Campbell et al., 2014). These standardized intervention programs are scientifically designed and are aimed at improving strength, proprioception, coordination and neuromuscular control. When properly implemented in youth soccer teams these interventions are efficacious in reducing lower limb injuries by 32–65% (Hübscher & Refshauge, 2013; Emery & Meeuwisse, 2010; LaBella et al., 2011; Soligard et al., 2010). Specifically, these interventions have a 77–90% risk reduction in knee injuries and 50–66% risk reduction in ankle injuries (Hübscher & Refshauge, 2013; Emery & Meeuwisse, 2010; LaBella et al., 2011; Kiani et al., 2010). Both acute and chronic injuries are reduced, and when an injury does occur it tends to be less severe (Emery & Meeuwisse, 2010; LaBella et al., 2011; Kiani et al., 2010; Soligard et al., 2008).

Despite the well-documented efficacy of these interventions in controlled situations, the small body of literature available on the subject suggests that uptake of these programs by real-world soccer teams in uncontrolled settings is poor (Emery & Meeuwisse, 2010; Soligard et al., 2008; Finch & Donaldson, 2010). One study conducted in Norway found that over half of Norwegian youth soccer coaches do not use an injury prevention program (Soligard et al., 2010). Even when teams do adopt such interventions, it appears that long-term compliance to the programs is not sustained. The outset of a randomized controlled trial conducted by Steffen et al. saw a 60% compliance rate among youth female soccer teams; unfortunately, compliance had dropped below 25% by the end of the study (Steffen et al., 2008). These data are similar to findings across other sports, including basketball and netball (Emery et al., 2007; Saunders et al., 2010). This suggests the presence of a barrier or discord in communication, knowledge, or intervention between the current literature and the sporting world as a whole (Alentorn-Geli et al., 2009). Stage 5 of Finch’s *Translating Research into Injury Prevention Practice* framework (TRIPP) calls for research on this gap between efficacy research and implementation. Studies must be done to understand the implementation context of interventions (Finch, 2006). Implementation context encompasses personal, environmental, societal and delivery factors that impede or promote uptake and adherence (Finch & Donaldson, 2010; Finch, 2006).

The current study seeks to better understand the coaching aspect of that implementation context as outlined by TRIPP Stage 5. Coaches have been chosen as the target research population because of the widespread consensus that coaches remain the most influential individuals for injury prevention in sports settings (Gardiner & Ranalli, 2000; Bell, 1992). Indeed, coaches are uniquely placed to teach safe play, promote prevention behaviors and make immediate decisions about injury management (Carter & Muller, 2008). In soccer specifically, the decision to incorporate or exclude an injury prevention program into practice and game plans lies primarily with the coach, therefore, the coach’s awareness and attitude toward such programs is a major determinant of whether interventions reach their player beneficiaries at all (Finch & Donaldson, 2010; White et al., 2014). It is becoming clear that all these efficacious interventions will not translate to a decrease in sports injuries if real-world teams do not adopt them and, more specifically, if coaches do not deliver them.

The purpose of this study is to determine (1) the level of awareness by youth coaches of these injury prevention programs and the efficacy of these programs in injury prevention; (2) the number of youth coaches that use these interventions; and (3) barriers and potential facilitators to implementing a sustainable injury prevention program such as coach knowledge, attitude, and perception of injuries and injury prevention.

## Methods

### Survey development

Ethics approval was acquired (McMaster Research Ethics Board - SREC#: 2013 77) and informed consent was obtained from all respondents. To our knowledge there are no validated questionnaires addressing soccer coach experience with injury prevention programs. Therefore, a survey to collect information on the perception of youth soccer injuries and awareness of injury prevention programs among coaches was developed. Question development was guided by the RE-AIM Sports Setting Matrix in combination with findings from the literature review and expert experience from a number of orthopaedic surgeons specializing in sport medicine (Finch & Donaldson, 2010). Pilot testing was performed prior to the beginning of the study to assess face validity of each question. The survey was distributed to 30 individuals who were felt to be representative of the final testing cohort due to their varying levels of soccer playing and coaching experience. Each question was rated for clarity, comprehension, and appropriateness on a scale of 1 to 5. Questions scoring an average below 4.0 on any of the parameters were to be discarded; however, all questions scored above 4.5 and were considered suitable for this study.

This survey consisted of 26 questions covering coaches’ demographics, level of training, experience with injuries among players, and use of injury prevention programs (Additional file 1). Questions that sought opinion-based answers were either yes/no or collected on a five-point Likert scale from “strongly disagree” to “strongly agree.” Questions about coach demographics were multiple choice or “indicate all that apply” options. In areas where the experts thought additional comments would contribute to our understanding of the answers, some questions also included the option of an open-ended response.

### Survey software and administration

The survey was uploaded to recognized online survey software, Survey Monkey. Respondents were provided with the link, and once a respondent had submitted the survey, they could not respond again. Results were compiled using the Survey Monkey software. All responses were voluntary and anonymous. Fifteen local soccer clubs were identified, and coaches of under 12 to under 18 teams were contacted through the club’s website information. Where possible, club presidents were asked to assist with contacting their coaches.

The email sent to the coaches outlined the purpose of the survey and provided the survey link. The survey was emailed to a total of 418 coaches, and a reminder email was sent to all coaches 3 weeks later. Respondents received no benefit, monetary or otherwise, from completing the survey.

### Statistical analysis

Responses were organized in Microsoft Excel 2010 and analyzed using Minitab 16. Descriptive statistics are presented as percentages compared using Chi-squared test. Percentages with 95% confidence interval (CI) are reported. A *p*-value of 0.05 was set for statistical significance.

It should be noted that some questions allowed free-text answers if respondents felt the options in the drop-down menu did not apply to them. The team determined that the free-text responses received did not add to the main purposes of the manuscript. For example, some coaches identified old or outdated coaching courses that were not available in the multiple choice answer set. These responses were therefore excluded where necessary for analysis.

## Results

One hundred and one of 418 coaches who were sent the survey responded for a response rate of 24.2% (95% CI, 20.0 to 28.5%). The sample size did not reach 101 for some questions as not all questions were answered by every coach and there were some answers that exceeded 101, presumably because some of the respondents coached multiple teams and were allowed to check multiple answers. Forty-seven percent (47.4%) of respondents coached “house league” soccer and half (49.5%) were aged 41–50. Seventy-five percent (75.3%, CI, 66.8 to 83.7%) of respondents had completed some level of coaching course. All coaches of Premier, Elite, and higher-level teams had completed some kind of coaching course, however, only 54.3% (56/101) of house league coaches reported completing coaching courses. Response rates decreased as the age of the team increased from U12 to U18 which likely reflects fewer teams in the older age groups. The results of the survey can be seen in Additional file 1.

### Awareness of injuries and injury interventions

Only one third of respondents agreed or strongly agreed that coaching courses had discussed the risk of injury for youth soccer players (34.8%), and the number was similar for coaching courses that had advocated for interventions (31.9%). Most coaches disagreed or strongly disagreed that their soccer clubs had been a source of injury awareness (52.6%) or advocated for the use of an intervention (52.1%). Awareness regarding injuries and prevention in youth players also came from incidents of personal injury, experience as a player, and from independent personal research.

The majority of coaches (80.6%) reported the occurrence of a non-season ending lower limb injury for a player on their team, and many had also seen a player on their team suffer a season-ending lower limb injury (34.3%). Table 1 shows the coach use of an intervention compared to their relationship with player injuries and there were no significant differences between those who used an intervention and those who did not.

**Table 1:** Injury incidence vs use of intervention

	A player on my team has suffered a season-ending lower limb injury Number (%)		A player on my team has suffered a lower limb injury that is not season-ending, but has caused them to miss one game or more Number (%)	
	Yes	No	Yes	No
Coach used intervention	7 (25.9%)	20	21 (75.0%)	7
Coach did not use intervention	15 (26.7%)	41	44 (78.5%)	12
*p*-value	0.460		0.784	

Tables 2 and 3 show the injury incidence and intervention use by coaches of male versus female teams respectively. There were no statistically significant differences between the incidence of injuries that were not season-ending or season ending for male versus female teams (*p* = 1.00). There were also no statistical differences in the use of an injury prevention program described by coaches of male versus female teams in the season preceding the questionnaire.

**Table 2:** Injury incidence of male and female teams

	Coaches of male teams Number (%)		Coaches of female teams Number (%)		*P*-value
	Yes	No	Yes	No	
	*n* = 43		*n* = 59		
A player on my team has suffered a season-ending lower limb injury	12 (28.0%)	31	23 (39.0%)	36	0.294
	*n* = 43		*n* = 60		
A player on my team has suffered a lower limb injury that is not season-ending, but has caused them to miss one game or more	35 (81.4%)	8	48 (80.0%)	12	1.00

**Table 3:** Intervention use by coaches of male and female teams

	Coaches of male teams Number^a^ (%)		Coaches of female teams Number^a^ (%)		*P*-value
	Agree or strongly agree	Disagree or strongly disagree	Agree or strongly agree	Disagree or strongly disagree	
I used an injury prevention program in the prior season	14 (35.9%)	25	17 (31.5%)	37	0.526

^a^Numbers exclude coaches that neither agreed nor disagreed with the statement

### Self-reported injury intervention use

The vast majority of coaches used a warm up with their teams before practices and games (92.6%), while only a fraction of that number used an injury prevention intervention in the prior season (29.8%). Only 25.0% of house league coaches used interventions versus 40.5% of coaches from Multijurisdictional/South region and 30.0% of coaches at even higher levels (Table 4). Injury prevention programs were used by 35.7% of coaches who had completed Community Coaching courses and 36.8% of coaches who had completed a higher-level course (Pre-B, Provincial B or National course) compared with 18.7% of coaches who had never completed any coaching courses, although this difference did not reach statistical significance (Table 5). Associated with a higher season-ending lower limb injury rate described by female coaches was a trend towards a lower use of an injury prevention program described by coaches of female teams in the season preceding the questionnaire, although this did not reach statistical significance (*p* = 0.526) (Table 3).

**Table 4:** Intervention use by level of team coached

	House league Number* (%)		Multijurisdictional or South Region (Divisions 1–4) Number* (%)		Premier, Elite, OYSL or OPDL Number* (%)	
	Agree or strongly agree	Disagree or strongly disagree	Agree or strongly agree	Disagree or strongly disagree	Agree or strongly agree	Disagree or strongly disagree
I used an injury prevention program in the prior season	9 (25.0%)**	27	13 (40.5%)	19	6 (30.0%)	14

*Numbers exclude coaches that neither agreed nor disagreed with the statement

**Percentages and *p*-value is calculated using total. *P* = 0.326

**Table 5:** Relationship between coaching course completion and intervention use

	None Number* (%)		One or more Community Coaching courses Number* (%)		Pre-B, Provincial B, National B, National A Number* (%)	
	Agree or strongly agree	Disagree or strongly disagree	Agree or strongly agree	Disagree or strongly disagree	Agree or strongly agree	Disagree or strongly disagree
I used an injury prevention program in the prior season	3 (18.7%)	13 (81.3%)	15 (35.7%)	27 (64.3%)	7 (36.8%)	12 (63.2%)

*Numbers exclude coaches that neither agreed nor disagreed with the statement

**Percentages are calculated using total. *P* = 0.418

### Barriers and facilitators to intervention use

Very few respondents cited too much time commitment as a reason for not using an intervention (6.6%). Of those that did not already use an intervention, coaches agreed or strongly agreed that they would consider using one if it could be used in place of warm up and would take no more than 20 min (74.0%), that they could access information about the exercises (84.0%), and if the exercises could be properly demonstrated (84.0%). Additionally, 84% of the coaches that did not already use an intervention agreed or strongly agreed that knowing that an intervention may reduce a player’s risk of injury by 45% would affect whether they would use an intervention.

### Other impressions

The final survey question asked for any additional commentary the respondents may have had, and these open responses elucidated a number of recurring concerns as seen in Additional file 1. Multiple respondents said that the limited practice time of house league players is a barrier for implementing interventions, as they tend to practice and play games less often. Finally, numerous coaches expressed the erroneous belief that most injuries result not from noncontact movements, but rather from dangerous, aggressive play and infractions involving contact or collisions between players.

## Discussion

Overall, coach awareness around injury prevention was highly variable. Perceived efficacy and accessibility of interventions were identified as potential facilitators to uptake, among other things; however, lack of information or misinformation about efficacy and accessibility were major barriers to uptake. Unfortunately, more than half of coaches reported that coaching courses or soccer clubs did not promote awareness around this topic. The most important finding in this study was that only 29.8% of respondents used an injury prevention intervention with their teams in the prior playing season, despite a convincing body of literature demonstrating the efficacy of neuromuscular training in injury prevention (Hübscher & Refshauge, 2013). This finding is more concerning as multiple studies have identified the coach as the pivotal intermediary in the implementation of injury prevention programs, and their motivation appears to correlate with player motivation in the participation of these interventions (Bizzini et al., 2013; Soligard et al., 2010). There has also been substantial scientific as well as mainstream media attention given to the female athlete and their apparent predisposition to ACL injuries, however, the current study failed to show coaches of female teams adopting an injury prevention intervention more so than coaches of male teams.

Bizzini et al. highlighted that education and communication from soccer associations and coaching clinics are important factors for increasing the use of interventions (Bizzini et al., 2013). Unfortunately, this study demonstrated that few clubs provide awareness regarding either risk of injury or injury prevention. Similarly, most coaches felt that soccer courses did not adequately discuss injury prevention interventions. In fact, 50% of coaches in the current study disagreed that the coaching course they attended advocated for the use of an injury prevention program or directed them to other resources to find information about such programs. Although we agree with Orr et al. that coaching courses should be encouraged across all levels of play in order to promote injury prevention (Orr et al., 2013), these courses must include specific information about injury prevention programs. Unfortunately, the current study reports that only 54% of house league coaches had completed a coaching course.

Coaches of the highest-level teams (Premier, Elite and higher), all of whom reported completing a coaching course, used interventions at a similar rate as those coaching lower level rep teams (Multijurisdictional and Div. 1–4 coaches). In part this may be due to the fact that not all courses taught injury prevention programs. In addition, McKay et al. found that players and coaches with more years of playing experience are less likely to adhere to injury prevention programs because they did not find the programs appropriate for the elite level, or their degree of experience rendered them confident in their alternate choice of warm-up and they were less willing to modify that choice (McKay et al., 2014).

There is evidence that the strongest motivator for coaches to use an injury prevention program is the knowledge that a reduction in the number of injuries will lead to a more successful team overall (Soligard et al., 2010). Frequent barriers to compliance with injury prevention programs include the belief that the interventions are too time-consuming, and that the interventions are not sufficiently soccer-specific (Soligard et al., 2010). Coach education, therefore, needs to emphasize the efficacy of injury prevention programs (Junge et al., 2002), especially when the literature suggests properly implemented interventions in youth soccer teams are efficacious in reducing lower limb injuries by 32–65% (Hübscher & Refshauge, 2013; Emery & Meeuwisse, 2010; LaBella et al., 2011; Soligard et al., 2010) and many of the major interventions (Prevent Injury and Enhance Performance Program, FIFA 11+) are readily available online at no cost, and take 20 minutes or less for completion (The PEP Program, 2011; FIFA 11+: a complete warm-up programme, 2011). Our results support this strategy as over 80% of the coaches in the current study that were not using an injury prevention intervention would consider using one knowing that it may reduce a player’s risk of injury by 45%, and almost three-quarters of respondents would consider using an intervention if it could be used in place of a warm up and take 20 minutes or less.

There is conflicting evidence regarding the relationship between skill level and rates of injury. Some studies have reported higher incidence of injury among elite players, possibly due to the intensity of play (Emery et al., 2005; Emery & Meeuwisse, 2006), however, other studies have reported that less-skilled teams tend to procure more injuries, and that these injuries are more severe than those of elite teams (Chomiak et al., 2000; Dvorak et al., 2000). More research needs to be done on this topic, however, the finding in this study that House league coaches used interventions less than coaches of higher level teams is concerning. This is likely the result of several factors including the fact that nearly half of house league coaches did not complete any sort of coaching course, the lack of time commitment from house league players with some arriving only a few minutes before the start of games, and that many house league teams are only allotted one hour of practice time per week.

### Study limitations

This was a cross-sectional study and is subject to the inherent bias of this type of research. For example, it is difficult to determine if coach beliefs preceded or anteceded the prior playing season. The response rate was low (24%) which could create a selection bias and meant that a number of subsets could not be effectively analyzed due to the relatively small respondent sample size.

Furthermore, the survey did not track which club the respondents belonged to as it was felt this would be a deterrent to club participation. Given this, it is possible that the results may represent a “club effect” if participants disproportionately responded from some clubs over others. Similarly, there are only a small number of coaches that coach more than one team and in order to maintain their anonymity no attempt was made to separate these few individuals out for a separate analysis.

## Conclusion

This study suggests that the current use and awareness of injury prevention programs is limited by a lack of communication and education between sporting associations and coaches, as well as perceived time constraints. In order to increase the frequency of intervention use, soccer clubs and coaching courses should focus on increasing the education of coaches targeting three main areas: reinforcing the efficacy of interventions; changing perceptions of the time required to perform these interventions; and providing access to intervention resources. Finally, coaching courses that emphasize intervention programs should be mandatory for all youth soccer coaches.

## Additional file

Additional file 1: Survey Questions and Results. (DOCX 31 kb)

## Abbreviations

ACL: Anterior cruciate ligament
TRIPP: Translating Research into Injury Prevention Practice
