# Supplementary material for: Lower limb injury prevention programs in youth soccer: a survey of coach knowledge, usage, and barriers
Source: J Exp Orthop. 2018 Oct 11;5:43. doi: 10.1186/s40634-018-0160-6 (PMC6179968; doi:10.1186/s40634-018-0160-6)
Supplement: Supplementary file 1 — Survey Questions and Results. (DOCX 31 kb) [file 40634_2018_160_MOESM1_ESM.docx]

**Additional file 1: Survey Questions and Results**

| 1. Please indicate your age category. | | | |
| --- | --- | --- | --- |
| Answer Options | Response Percent | | Response Count |
| 18-30 | 16.8% | | 17 |
| 31-40 | 19.8% | | 20 |
| 41-50 | 49.5% | | 50 |
| 51-60 | 10.9% | | 11 |
| >60 | 3.0% | | 3 |
| *answered question* | | | 101 |
| *skipped question* | | | 0 |
|  | | | |
| 2. Please indicate the age group and gender of the teams you coach. Check all that apply. (M=male; F=Female) | | | |
| Answer Options | Response Percent | | Response Count |
| U12M | 20.4% | | 20 |
| U12F | 19.4% | | 19 |
| U13M | 10.2% | | 10 |
| U13F | 13.3% | | 13 |
| U14M | 10.2% | | 10 |
| U14F | 12.2% | | 12 |
| U15M | 9.2% | | 9 |
| U15F | 11.2% | | 11 |
| U16M | 6.1% | | 6 |
| U16F | 11.2% | | 11 |
| U17M | 2.0% | | 2 |
| U17F | 4.1% | | 4 |
| U18M | 6.1% | | 6 |
| U18F | 8.2% | | 8 |
| Other (please specify) | 16.3% | | 16 |
| *answered question* | | | 98 |
| *skipped question* | | | 3 |
|  | | | |
| 3. What level does your team compete at? | | | |
| Answer Options | Response Percent | | Response Count |
| House league | 47.4% | | 46 |
| Multijurisdictional | 13.4% | | 13 |
| Division 4 | 5.2% | | 5 |
| Division 3 | 4.1% | | 4 |
| Division 2 | 11.3% | | 11 |
| Division 1 | 8.2% | | 8 |
| Premier | 14.4% | | 14 |
| Elite | 9.3% | | 9 |
| OYSL | 1.0% | | 1 |
| Other (please specify) | 6.2% | | 6 |
| *answered question* | | | 97 |
| *skipped question* | | | 4 |
|  | | | |
| 4. The highest level of coaching courses you have successfully completed is: | | | |
| Answer Options | Response Percent | | Response Count |
| None | 24.7% | | 24 |
| One or more Community Coaching courses | 45.4% | | 44 |
| Pre B assessment | 8.2% | | 8 |
| Provincial B license | 6.2% | | 6 |
| National B license | 5.2% | | 5 |
| A license | 2.1% | | 2 |
| Other (please specify) | 8.2% | | 8 |
| *answered question* | | | 97 |
| *skipped question* | | | 4 |
|  | | | |
| 5. Are you in a profession related to health care? | | | |
| Answer Options | Response Percent | | Response Count |
| No | 86.6% | | 84 |
| Yes (please specify) | 13.4% | | 13 |
| *answered question* | | | 97 |
| *skipped question* | | | 4 |
|  | | | |
| 6. Has a player on your team ever suffered a season-ending lower limb injury? | | | |
| Answer Options | Response Percent | | Response Count |
| No | 71.9% | | 69 |
| Yes | 28.1% | | 27 |
| *answered question* | | | 96 |
| *skipped question* | | | 5 |
|  | | | |
| 7. Has a player on your team ever suffered a lower limb injury that is not season-ending, but has caused them to miss one game or more? | | | |
| Answer Options | Response Percent | | Response Count |
| No | 23.7% | | 23 |
| Yes | 76.3% | | 74 |
| *answered question* | | | 97 |
| *skipped question* | | | 4 |
|  | | | |
| 8. The coaching courses I have taken have discussed the risk of lower limb injuries for young soccer players. | | | |
| Answer Options | Response Percent | | Response Count |
| Strongly disagree | 9.5% | | 9 |
| Disagree | 20.0% | | 19 |
| Neither agree nor disagree | 35.8% | | 34 |
| Agree | 31.6% | | 30 |
| Strongly agree | 3.2% | | 3 |
| *answered question* | | | 95 |
| *skipped question* | | | 6 |
|  | | | |
| 9. The soccer club where I coach has promoted awareness about the risk of lower limb injuries for soccer players, either in meetings, emails, or by other means. | | | |
| Answer Options | Response Percent | | Response Count |
| Strongly disagree | 10.5% | | 10 |
| Disagree | 42.1% | | 40 |
| Neither agree nor disagree | 23.2% | | 22 |
| Agree | 18.9% | | 18 |
| Strongly agree | 5.3% | | 5 |
| *answered question* | | | 95 |
| *skipped question* | | | 6 |
|  | | | |
| 10. Do you have any other experiences that would contribute to your awareness of lower limb injuries in soccer players? | | | |
| Answer Options | Response Percent | | Response Count |
| No | 37.9% | | 36 |
| Yes (please specify) | 62.1% | | 59 |
| *answered question* | | | 95 |
| *skipped question* | | | 6 |
|  | | | |
| 11. I currently use a warm up for my team before practices and games. | | | |
| Answer Options | Response Percent | | Response Count |
| Strongly disagree | 3.2% | | 3 |
| Disagree | 2.1% | | 2 |
| Neither agree nor disagree | 2.1% | | 2 |
| Agree | 26.3% | | 25 |
| Strongly agree | 66.3% | | 63 |
| *answered question* | | | 95 |
| *skipped question* | | | 6 |
|  | | | |
| 12. I have attended a coaching course that has advocated for the use of an injury prevention program for my team, or directed me to other resources to find information about such programs. | | | |
| Answer Options | Response Percent | | Response Count |
| Strongly disagree | 16.0% | | 15 |
| Disagree | 34.0% | | 32 |
| Neither agree nor disagree | 18.1% | | 17 |
| Agree | 23.4% | | 22 |
| Strongly agree | 8.5% | | 8 |
| *answered question* | | | 94 |
| *skipped question* | | | 7 |
|  | | | |
| 13. I have attended a coaching course that has demonstrated the exercises of an injury prevention program. | | | |
| Answer Options | Response Percent | | Response Count |
| Strongly disagree | 11.8% | | 11 |
| Disagree | 34.4% | | 32 |
| Neither agree nor disagree | 20.4% | | 19 |
| Agree | 25.8% | | 24 |
| Strongly agree | 7.5% | | 7 |
| *answered question* | | | 93 |
| *skipped question* | | | 8 |
|  | | | |
| 14. My soccer club has advocated for the use of an injury prevention program for my team, or directed me to other resources to find information about such programs. | | | |
| Answer Options | Response Percent | | Response Count |
| Strongly disagree | 11.7% | | 11 |
| Disagree | 40.4% | | 38 |
| Neither agree nor disagree | 23.4% | | 22 |
| Agree | 17.0% | | 16 |
| Strongly agree | 7.4% | | 7 |
| *answered question* | | | 94 |
| *skipped question* | | | 7 |
|  | | | |
| 15. My club or a club representative has demonstrated how to perform an injury prevention program to my team or me. | | | |
| Answer Options | Response Percent | | Response Count |
| Strongly disagree | 19.1% | | 18 |
| Disagree | 44.7% | | 42 |
| Neither agree nor disagree | 17.0% | | 16 |
| Agree | 16.0% | | 15 |
| Strongly agree | 3.2% | | 3 |
| *answered question* | | | 94 |
| *skipped question* | | | 7 |
|  | | | |
| 16. Do you have any other experiences that would contribute to your awareness of injury prevention programs (ie. advertisements, personal research, etc.)? | | | |
| Answer Options | Response Percent | | Response Count |
| No | 58.5% | | 55 |
| Yes (please specify) | 41.5% | | 39 |
| *answered question* | | | 94 |
| *skipped question* | | | 7 |
|  | | | |
| 17. I have used an injury prevention program in 2013. | | | |
| Answer Options | Response Percent | | Response Count |
| Strongly disagree | 12.8% | | 12 |
| Disagree | 46.8% | | 44 |
| Neither agree nor disagree | 10.6% | | 10 |
| Agree | 12.8% | | 12 |
| Strongly agree | 17.0% | | 16 |
| *answered question* | | | 94 |
| *skipped question* | | | 7 |
|  | | | |
| 18. I have used an injury prevention program prior to 2013. | | | |
| Answer Options | Response Percent | | Response Count |
| Strongly disagree | 13.8% | | 13 |
| Disagree | 41.5% | | 39 |
| Neither agree nor disagree | 9.6% | | 9 |
| Agree | 21.3% | | 20 |
| Strongly agree | 13.8% | | 13 |
| *answered question* | | | 94 |
| *skipped question* | | | 7 |
|  | | | |
| 19. I plan to use an injury prevention program in the upcoming season. | | | |
| Answer Options | Response Percent | | Response Count |
| Strongly disagree | 1.1% | | 1 |
| Disagree | 18.5% | | 17 |
| Neither agree nor disagree | 32.6% | | 30 |
| Agree | 27.2% | | 25 |
| Strongly agree | 20.7% | | 19 |
| *answered question* | | | 92 |
| *skipped question* | | | 9 |
|  | | | |
| 20. The reason I do not use an injury prevention program with my team is because it takes up too much of the practice time. | | | |
| Answer Options | Response Percent | | Response Count |
| Strongly disagree | 18.7% | | 17 |
| Disagree | 30.8% | | 28 |
| Neither agree nor disagree | 29.7% | | 27 |
| Agree | 6.6% | | 6 |
| Strongly agree | 0.0% | | 0 |
| Not applicable (I already use an injury prevention program) | 14.3% | | 13 |
| *answered question* | | | 91 |
| *skipped question* | | | 10 |
|  | | | |
| 21. I would consider using an injury prevention program if it could be used in place of warm up and would take no more than 20 minutes. | | | |
| Answer Options | Response Percent | | Response Count |
| Strongly disagree | 2.2% | | 2 |
| Disagree | 3.3% | | 3 |
| Neither agree nor disagree | 15.2% | | 14 |
| Agree | 46.7% | | 43 |
| Strongly agree | 12.0% | | 11 |
| Not applicable (I already use an injury prevention program) | 20.7% | | 19 |
| *answered question* | | | 92 |
| *skipped question* | | | 9 |
|  | | | |
| 22. The reason I do not use an injury prevention program with my team is because I do not know the exercises. | | | |
| Answer Options | Response Percent | | Response Count |
| Strongly disagree | 3.3% | | 3 |
| Disagree | 16.3% | | 15 |
| Neither agree nor disagree | 14.1% | | 13 |
| Agree | 38.0% | | 35 |
| Strongly agree | 7.6% | | 7 |
| Not applicable (I already use an injury prevention program) | 20.7% | | 19 |
| *answered question* | | | 92 |
| *skipped question* | | | 9 |
|  | | | |
| 23. I would consider using an injury prevention program if I could access information about the exercises. | | | |
| Answer Options | Response Percent | | Response Count |
| Strongly disagree | 1.1% | | 1 |
| Disagree | 0.0% | | 0 |
| Neither agree nor disagree | 11.1% | | 10 |
| Agree | 52.2% | | 47 |
| Strongly agree | 12.2% | | 11 |
| Not applicable (I already use an injury prevention program) | 23.3% | | 21 |
| *answered question* | | | 90 |
| *skipped question* | | | 11 |
|  | | | |
| 24. I would consider using an injury prevention program if somebody demonstrated how to do the exercises properly. | | | |
| Answer Options | Response Percent | | Response Count |
| Strongly disagree | 1.1% | | 1 |
| Disagree | 0.0% | | 0 |
| Neither agree nor disagree | 11.2% | | 10 |
| Agree | 56.2% | | 50 |
| Strongly agree | 9.0% | | 8 |
| Not applicable (I already use an injury prevention program) | 22.5% | | 20 |
| *answered question* | | | 89 |
| *skipped question* | | | 12 |
|  | | | |
| 25. The knowledge that injury prevention programs may reduce a player’s risk of injury by 45% affects whether I would use an injury prevention program with my team. | | | |
| Answer Options | | Response Percent | Response Count |
| Strongly disagree | | 0.0% | 0 |
| Disagree | | 1.1% | 1 |
| Neither agree nor disagree | | 11.2% | 10 |
| Agree | | 43.8% | 39 |
| Strongly agree | | 22.5% | 20 |
| Not applicable (I already use an injury prevention program) | | 21.3% | 19 |
| *answered question* | | | 89 |
| *skipped question* | | | 12 |
| \| 26. If you have any other comments about injuries and injury prevention in soccer, or you would like to elaborate on any of your answers, please use the space below. \| \| --- \| | | | |
| Answers: | | | |
| Injuries in Soccer are more common due to infractions opposed to personal injury. My years of coaching have seen players in excellent condition and take the precautions to prevent injuries but many of officials allow fouls to continue and only when it reaches an injury they try to be more enforced. | | | |
| Injury prevention programs should be part of Standard coaching information sessions or courses. | | | |
| Mechanics are important when performing the tasks. Players can take short cuts. For example, rather than make sharp turns, they will round them out. The net effect is that they are not getting the full benefit of the exercises. Coaches must demonstrate and correct. | | | |
| Sports injury prevention would be very beneficial for players and parents. | | | |
| It has never really been an issue at the house league level. We almost need to assume or encourage the players to arrive to games or practices already warmed up. | | | |
| Sorry, the overall impression i would want to leave (as i think i misunderstood some earlier questions) is that i am not aware of an actual injury prevention program...i am aware of higher risks associated with female athletes and some exercises to help prevent these injuries . I would absolutely welcome a 20min program that would target these risks and be in place or part of a dynamic warm up. | | | |
| Injuries I’ve seen are not due to lack of warmup, mostly collisions. | | | |
| Hi. Been coaching kids for the past 9 years and as the kids have become older, I have implemented more pregame and practice stretching. I disagreed with the 20 minute program because 10 minutes should be more than enough time. They injury prevention that needs to be taught more revolves around dangerous play. I have seen many sprained ankles as a result of slide tackling and many cut calves from the cleats of the shoes. there are many more injuries caused from this than hamstring pulls for the kids I've coached. | | | |
| In House League, players show up 5 minutes before the game, won't come to practice-it's tough to fit the time in when players and parents won't buy into the commitment to play | | | |
| The Ontario soccer association does not promote such awareness to its District, or club lebel associations. It does however use such awareness as part of the regional and provincial programs. The newly launched OPDL program neglects to really focus on the issue in particular for young female players who may be prone to a higher incidence of knee related issues (depends on which research you tend to gravitate to). Much more programming is necessary and technical directors within programs should play the role of disseminating information, techniques etc. to new coaches within their programs. It’s never too late to start its never too late to learn more. | | | |
| I always do pre / post exercises to help reduce but it is not under any formal program. Also, the exercises I do could be optimized by incorporating a formal program. Unfortunately many injuries also occur purely due to field conditions. | | | |
| For a house league team the practice is only one hour per week, which already is not enough to really teach; and for games players often arrive just minutes before the game. So as much as I agree with the usefulness of an injury prevention program, there just is not enough time. | | | |
| I find, not only warm up stretches to be desirable, but also a common sense approach, coaching restraint, not being provoked and use of fairness while playing, endorses safety for all. In my experience over the years I have seen players acting rough, with the "okay" of their coaches, as the main culprit. | | | |
| ACL injuries in female soccer players is extremely common and most players and coaches are aware of this however I have not heard of or experienced any injury prevention programs. I am also not aware if any programs are successful or not. There has also been little to no discussion during coach seminars etc. | | | |
| I find that many lower limb injuries occur on artificial turf and I believe it is due to the fact that the turf does not allow the player's foot to "slide" through as effortlessly as on grass, causing ankle and knee injuries if the player tends to "drag" their feet rather than picking them up. Most, if not all injuries I have experienced occur on turf fields and many are to the same players over and over. | | | |
| *answered question* | | | 16 |
| *skipped question* | | | 85 |
